# Differential Detection of the Tobamoviruses Tomato Mosaic Virus (ToMV) and Tomato Brown Rugose Fruit Virus (ToBRFV) Using CRISPR-Cas12a

**DOI:** 10.3390/plants10061256

**Published:** 2021-06-21

**Authors:** Dan Mark Alon, Hagit Hak, Menachem Bornstein, Gur Pines, Ziv Spiegelman

**Affiliations:** 1 Department of Entomology, Agricultural Research Organization—the Volcani Center, 68 HaMaccabim Road, P.O. Box 15159, Rishon LeZion 7505101, Israel; alondanm@gmail.com; 2 The Shmunis School of Molecular Cell Biology & Biotechnology, Faculty of Life Science, Tel Aviv University, Tel Aviv 69978, Israel; menachemb@volcani.agri.gov.il; 3 Department of Plant Pathology and Weed Research, Agricultural Research Organization—the Volcani Center, 68 HaMaccabim Road, P.O. Box 15159, Rishon LeZion 7505101, Israel; hagith@volcani.agri.gov.il

**Keywords:** CRISPR/Cas12a, *Solanum lycopersicum*, *Tobamovirus*, ToBRFV, tomato brown rugose fruit virus, tomato mosaic virus, ToMV

## Abstract

CRISPR/Cas12a-based detection is a novel approach for the efficient, sequence-specific identification of viruses. Here we adopt the use of CRISPR/Cas12a to identify the tomato brown rugose fruit virus (ToBRFV), a new and emerging tobamovirus which is causing substantial damage to the global tomato industry. Specific CRISPR RNAs (crRNAs) were designed to detect either ToBRFV or the closely related tomato mosaic virus (ToMV). This technology enabled the differential detection of ToBRFV and ToMV. Sensitivity assays revealed that viruses can be detected from 15–30 ng of RT-PCR product, and that specific detection could be achieved from a mix of ToMV and ToBRFV. In addition, we show that this method can enable the identification of ToBRFV in samples collected from commercial greenhouses. These results demonstrate a new method for species-specific detection of tobamoviruses. A future combination of this approach with isothermal amplification could provide a platform for efficient and user-friendly ways to distinguish between closely related strains and resistance-breaking pathogens.

## 1. Introduction

Plant viruses of the *Tobamovirus* genus (family: *Virgaviridae*) are important crop pathogens, causing significant damage to the global agriculture industry [1,2,3]. The *Tobamovirus* genus contains several Solanaceae-infecting species, including the tobacco mosaic virus (TMV) and the tomato mosaic virus (ToMV). Tobamovirus particles are rod-shaped, encapsulating a single-stranded sense RNA (+ssRNA) genome of approximately 6.4 kb encoding four ORFs. ORFs 1 and 2 encode the two subunits of the viral replicase complex, separated by a read-through stop codon. ORF3 encodes the viral 30 kDa movement protein (MP). ORF4 encodes the 17–18 kDa viral coat protein (CP) [4,5]. Tobamoviruses are highly infectious and transmitted by mechanical contact with working hands, tools, soil, and parts of infected plants [6,7,8]. Importantly, tobamoviruses are also transmitted by seeds [9,10].

Tomato brown rugose fruit virus (ToBRFV), a new tomato-infecting tobamovirus emerged in tomato greenhouses in Israel and Jordan in 2014 [11,12]. ToBRFV was found to overcome all tobamovirus resistance genes in tomatoes, including the durable *Tm-2^2^* resistance gene, which has remained unbroken for over 60 years [12,13]. Outbreaks of ToBRFV in Europe [14,15,16,17,18,19], North America [20,21], and Asia [22,23], indicate a rapidly emerging global epidemic.

Serological methods such as enzyme-linked immunosorbent assay (ELISA) and Western-blot are currently used to detect ToBRFV [12,24,25,26]. However, these methods lack species-specificity and cannot be used to distinguish between ToBRFV and closely related tobamoviruses such as TMV and ToMV. This is due to the high conservation of the tobamovirus CP, resulting in antibody cross-reactivity between tobamovirus species [12]. To cope with this challenge, protocols for specific detection of ToBRFV were developed, including deep sequencing [12], sequence-specific reverse-transcription polymerase chain reaction (RT-PCR) primers [12,14,27,28,29], real-time RT-PCR [18,21,29,30] and loop-mediated isothermal amplification (LAMP) [30,31].

An emerging approach for the species-specific identification of viruses is based on CRISPR/Cas technology. The CRISPR/Cas is an innate immune system present in many bacteria and archaea [32]. In this system, Cas proteins target specific viral sequences by the formation of a ribonucleoprotein (RNP) complex with a CRISPR RNA (crRNA) complementary to the invading viral DNA sequence [33]. Directed by their crRNA, Cas proteins then bind and cleave the viral DNA, thereby neutralizing the pathogen [34]. While this system is mostly utilized for genome editing [35,36,37,38], more recently, it has been shown that some Cas proteins can be used for the detection of specific nucleic acid sequences [39,40,41]. For example, Cas12a obtains a nonspecific single-stranded DNAse activity following specific interaction with its DNA substrate [39]. This property was used by Chen and colleagues to apply Cas12a as a biosensor: in addition to the RNP complex of *Lachnospiraceae bacterium* Cas12a and its crRNA, a fluorophore quencher (FQ)–labeled single-stranded DNA substrate was added. Upon Cas12a activation, it nonspecifically degrades ssDNA, releasing the fluorophore from its quencher, resulting in a fluorescent signal. This approach was performed for the detection of human papillomavirus and has also proven efficient in severe acute respiratory syndrome coronavirus 2 (SARS-Cov-2) detection [39,42], demonstrating the great potential of this method in viral diagnostics.

**Figure 1:**
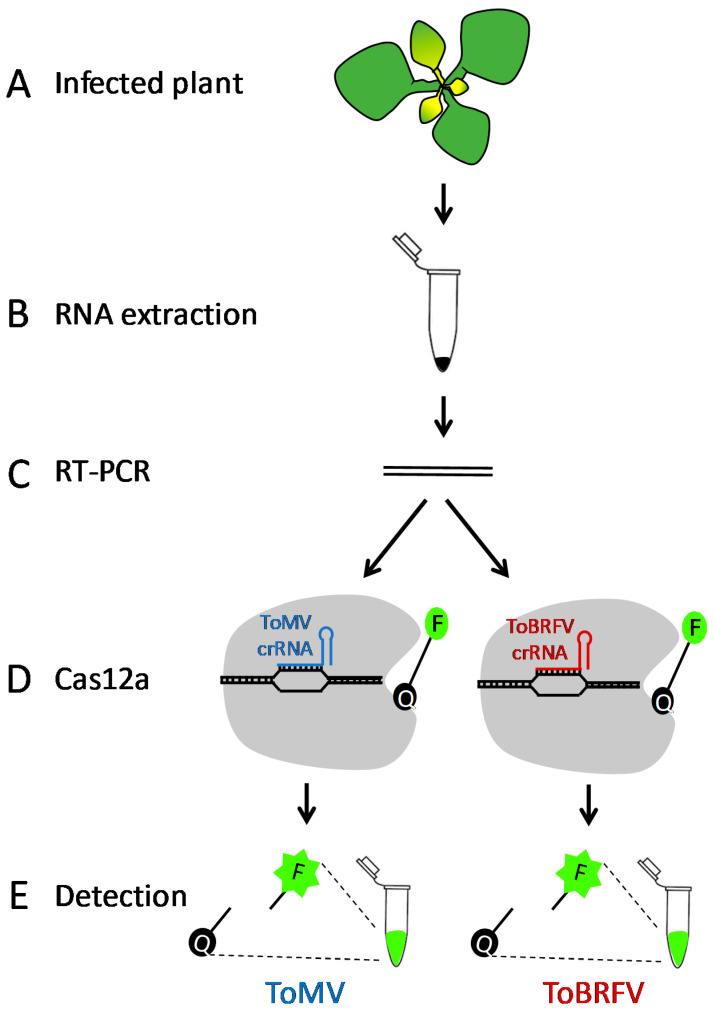
Illustration of plant virus detection using CRISPR/Cas12. (**A**) Leaf samples are collected from the infected plant. (**B**), RNA is extracted from the infected leaf. (**C**) Amplification of part of the viral genome using genus-specific RT-PCR primers. (**D**) Using species-specific crRNA, Cas12a-crRNAs specifically target the sequence of ToBRFV or ToMV. (**E**) Activation of Cas12a by sequence-specific binding, which triggers the degradation of the ssDNA probe and releases the fluorophore (F) from the quencher (Q) to emit the fluorescent signal.

Recently, CRISPR/Cas12a was applied for the detection of RNA [43,44] and DNA [45] plant viruses, suggesting its potential application in the detection of plant viral diseases. Here we adopt the CRISPR/Cas12a technology to specifically identify the emerging ToBRFV and to distinguish it from a closely related tobamovirus, ToMV. Moreover, we show that this method can be used to detect ToBRFV in samples from commercial greenhouses. Since TMV only rarely infects tomato plants [46], it was not included in this study. We have set up an experimental system based on the conservation and the variation between these two viruses (Figure 1). First, tomato plants were infected with each of the two viruses (Figure 1A). Then, RNA was extracted from leaves of the infected plant (Figure 1B) and RT-PCR was performed using primers on a conserved region from both viruses (Figure 1C). The resulting PCR product was then subjected to CRISPR/Cas12a detection assays using crRNAs specific for either ToMV or ToBRFV (Figure 1D). Cleavage of the ssDNA reporter and release of the fluorophore from the quencher indicates the presence of the specific virus (Figure 1E).

## 2. Materials and Methods

### 2.1. Plant Materials and Virus Inoculation

Tomato (*Solanum lycopersicum* L. cv. Moneymaker) (LA2706) were grown in soil in a light- and temperature-controlled chamber at 25 °C with a 16 h light/8 h dark regime. Three-week-old tomato plants were used for mechanical inoculation of ToMV and ToBRFV. ToMV was derived from the infectious clone pTLW3 [13]. For ToBRFV infection we used the Israeli isolate of ToBRFV, ToBRFV-IL (KX619418.1). Plants were dusted with carborundum powder prior to inoculation, and were then rubbed with a phosphate buffer solution (0.01 M, pH 7.0) supplemented with crushed leaves from ToMV- or ToBRFV-infected tomato plants. After three weeks, samples were collected from young leaves showing viral symptoms, distant from the site of inoculation.

For field sample analysis, tomato leaves with mosaic symptoms were collected from two commercial greenhouses in Azriel village, Israel, in December 2020.

### 2.2. RT-PCR Analysis

**Figure 2:**
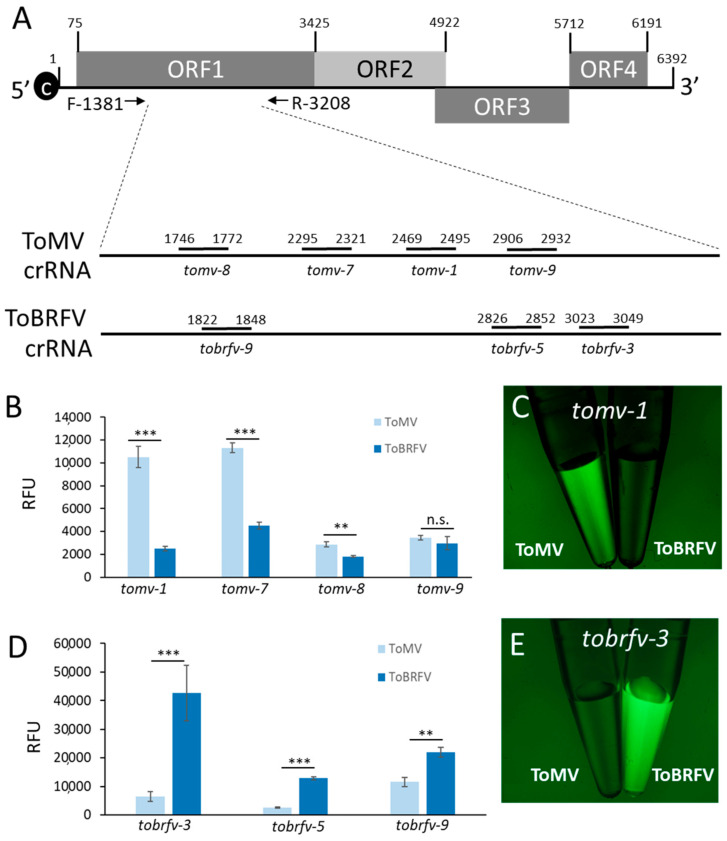
Analysis of different crRNAs for the detection of ToMV and ToBRFV. (**A**) A 1827 bp fragment was amplified using RT-PCR from virus-infected plants. Four crRNAs were designed for ToMV detection (*tomv-1*, *tomv-7*, *tomv-8* and *tomv-9*) and three sgRNAs were designed for ToBRFV detection (*tobrfv-3*, *tobrfv-5* and *tobrfv-9*). (**B**) CRISPR/Cas12-based fluorescent detection of ToMV using the different crRNAs on samples from ToMV- (light blue) and ToBRFV- (dark blue) infected tomato plants. (**C**) Fluorescence image of ToMV detection using the *tomv-1* crRNA. (**D**) CRISPR/Cas12-based fluorescent detection of ToBRFV using the different crRNAs on samples from ToMV (light blue) and ToBRFV (dark blue) infected tomato plants. (**E**) Fluorescence image of ToBRFV detection using the *tomv-3* crRNA. RFU = relative fluorescence units. n.s = not significant. ** *p* ≤ 0.01, *** *p* ≤ 0.001 in Student’s *t*-test.

Total plant RNA was extracted from 50–100 mg of leaf tissue by Plant Total RNA Mini Kit (Geneaid, RPD050, New Taipei City, Taiwan R.O.C), which includes a DNAse treatment. The extracted RNA (500 ng) served as a template for cDNA synthesis using the qPCRBIO cDNA Synthesis Kit, according to the manufacturer’s protocol (PCRBIO, PB30.11). The ToMV/ToBRFV ORF1 region (Figure 2A) was amplified using the Q5 High-Fidelity 2X Master Mix (New England Biolabs; NEB, Ipswich, MA, USA) with primers directed against conserved regions in both ToMV and ToBRFV F-1381 (5′-ccaggtctgagtgggatg-3′), and R-3208 (5′-gtctcaccttgtacctcatgtac-3′). PCR products were purified using Zymo DNA Clean & Concentrator kit (Zymo Research, Irvine, CA, USA), quantified using Nanodrop (Thermo Scientific, Waltham, MA, USA) and diluted to target concentrations using ultra-pure nuclease-free water.

### 2.3. CRISPR RNA (crRNA) Design

CRISPR RNA (crRNAs) (Table 1) were designed using the CRISPOR computational platform for the detection of efficient and specific design of crRNA sequences [47]. Both ToMV and ToBRFV genomes were obtained from the NCBI website (AF332868.1 and KX619418.1, respectively). To create unique crRNAs for each virus, the sequences of both viruses were concatenated into a single long sequence. This single sequence, harboring both viruses, was used as an input for CRISPOR guide gRNA search, allowing the detection of crRNAs that are specific to each virus (Figure 2A). The resulting crRNA sequences (Table 1) were then concatenated to a T7 promoter (T7-crRNA-F; Table 1) sequence and the designed oligonucleotides were ordered from Integrated DNA Technologies (IDT; https://eu.idtdna.com/, Coralville, USA) (T7-crRNA-F).

### 2.4. In Vitro crRNA Transcription

First, each DNA oligo was annealed with oligo T7-crRNA-F. Oligos were suspended in annealing buffer (10 mM Tris pH 8, 50 mM NaCl, 1 mM EDTA) to a final concentration of 100 uM. Next, 10 uL of each oligo was mixed with 10 uL T7-crRNA-F and incubated for 5 min at 95 °C and cooled at room temperature for 1 h. After oligo duplexes were ready, RNA was generated using TranscriptAid T7 High Yield Transcription Kit (Thermo Scientific) according to manufacturer protocol with overnight incubation. Subsequently, RNA was purified using Zymo RNA Clean & Concentrate kit (Zymo), with a DNase I digestion preceding the purification as suggested by the manufacturer’s protocol. RNA was diluted 1:150 and measured using a NanoDrop One instrument (Thermo Scientific).

### 2.5. LbCas12a Cleavage Assays

All reactions were prepared on ice. crRNA-LbCas12a complexes were prepared by mixing 62.5 nM gRNA with 50 nM LbCas12a (NEB) in NEBuffer 2.1 (NEB) to a final volume of 20 uL and incubated in 37 °C for 30 min. Next, 1 uM FAM reporter (/56-FAM/TTATTATT/3BHQ_1/)) and 1 nM (120 ng) RT-PCR product were added to the complexes together with 80 uL of 1X concentration NEBuffer 2.1 and incubated for 10 min at 37 °C. Samples were transferred to a black 384 well plate and measured using Tecan Spark plate-reader (Tecan Trading AG, Männedorf, Switzerland) with an excitation wavelength of 485 nm, and emission was measured at 535 nm. Emission was read in relative fluorescence units (RFU) to compare between samples within each experiment.

## 3. Results

### 3.1. Differential Detection of ToMV and ToBRFV Using CRISPR/Cas12a

A region within the tobamovirus ORF1 was used for the design of species-specific crRNAs (Figure 2A). Within this region, four ToMV-specific crRNAs (*tomv-1*, *tomv-7*, *tomv-8,* and *tomv-9*) and three ToBRFV-specific crRNAs (*tobrfv-3*, *tobrfv-5,* and *tobrfv-9*), were designed. To test the ability of the different crRNAs to detect ToMV or ToBRFV, 3-week-old tomato plants (*Solanum lycopersicum* L. cv. Moneymaker) were inoculated separately with ToMV and ToBRFV (*n* = 4). After three weeks, RNA was extracted from newly emerged systemic leaves showing viral symptoms. The different crRNA were then tested to specifically identify each virus in the CRISPR/Cas12a fluorescence assay. For ToMV detection, all four *tomv* crRNA-Cas12a complexes were incubated individually with the ToMV RT-PCR product. The ToBRFV RT-PCR product served as a negative control (Figure 2B). Among these crRNAs, *tomv-1* and *tomv-7* emitted robust fluorescent signals in response to ToMV in three independent experiments (*n* = 4), which were 4.2- and 2.5- times higher than with the ToBRFV negative control (Figure 2B,C). The *tomv-8* crRNA produced a fluorescent signal 57% higher than the control, and *tomv-9* signal was similar in ToMV and ToBRFV (Figure 2B) (*n* = 4).

A reciprocal experiment was performed for ToBRFV detection, only this time with the ToMV RT-PCR product serving as a negative control (Figure 2D). Here, *tobrfv-3* produced a strong fluorescent signal when incubated with the ToBRFV template, which was 6.5 times higher than the ToMV control template (Figure 2D,E) (*n* = 4). The *tobrfv-5* and *tobrfv-9* crRNAs were also efficient in ToBRFV detection, producing fluorescent signals that were 5 and 1.9 higher than the ToMV control (Figure 2D) (*n* = 4). These results were consistent in three independent experiments and established the ability of the CRISPR/Cas12a for species-specific identification of ToMV and ToBRFV. As the most efficient detection of ToMV and ToBRFV was achieved by the *tomv-1* and *tobrfv-3* crRNAs, respectively, these crRNAs were selected for further analysis.

### 3.2. Sensitivity and Specificity of CRISPR/Cas12a-Based Detection

**Figure 3:**
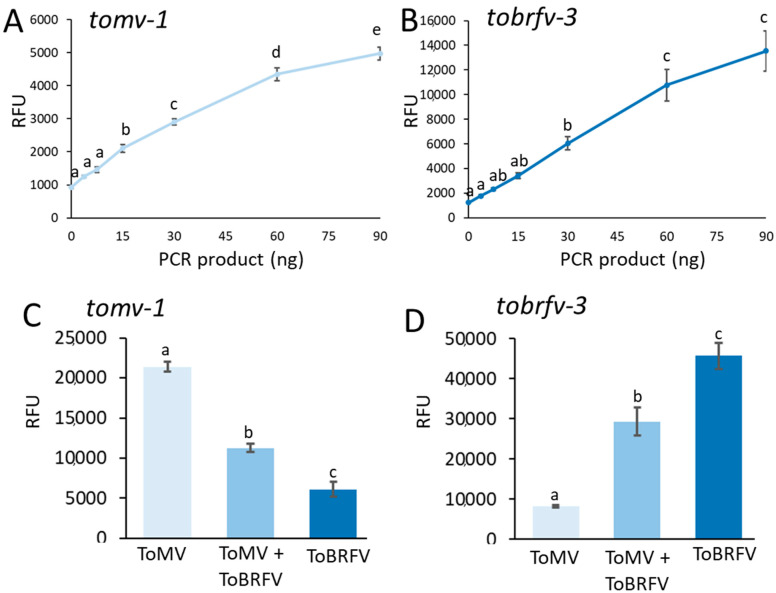
Sensitivity and specificity analysis for the selected crRNAs. (**A**) Detection of ToMV using the *tomv-1* crRNA in a series of dilutions of ToMV RT-PCR products. (**B**) Detection of ToBRFV using the *tobrfv-3* crRNA in a series of dilutions of ToBRFV RT-PCR products. (**C**) Identification of ToMV in a mix of ToMV and ToBRFV RT-PCR products from using the *tomv-1* crRNA. (**D**) Identification of ToBRFV in a mix of ToMV and ToBRFV RT-PCR products using the *tobrfv-3* crRNA. Different letters indicate statistical significance in Tukey-HSD test (*p* ≤ 0.05).

To test the sensitivity of this system, a series of dilutions was performed for each of the ToMV and ToBRFV RT-PCR products (Figure 3A,B). While 15 ng of RT-PCR product was sufficient for detecting ToMV (Figure 3A), 15–30 ng of RT-PCR product was needed for the detection of ToBRFV (Figure 3B). As 15–30 ng of PCR products was the minimal threshold for detection, we further used these parameters to examine if the CRISPR/Cas12a system could specifically detect each virus in the case of a mixture of ToMV and ToBRFV. To test this, detection assays were performed individually using *tomv-1* or *tobrfv-3* on a mixture of RT-PCR products from both ToMV and ToBRFV (Figure 3C,D). Notably, 15 ng of ToMV was detected in a mixture with 15 ng of ToBRFV, resulting in a fluorescent signal that was 85% higher than the signal obtained with 30 ng of ToBRFV only (Figure 3C). In addition, the CRISPR/Cas12a system was also able to detect 15 ng of ToBRFV mixed with 15 ng of ToMV, producing a fluorescent signal that was 3.5-fold higher than the signal received with only 30 ng of ToMV (Figure 3D). These results suggest that the CRISPR/Cas12a system can be used to detect ToBRFV, even in the case of mixed infection with the related virus ToMV.

### 3.3. CRISPR/Cas12a-Based Detection of ToBRFV from Field Samples

**Figure 4:**
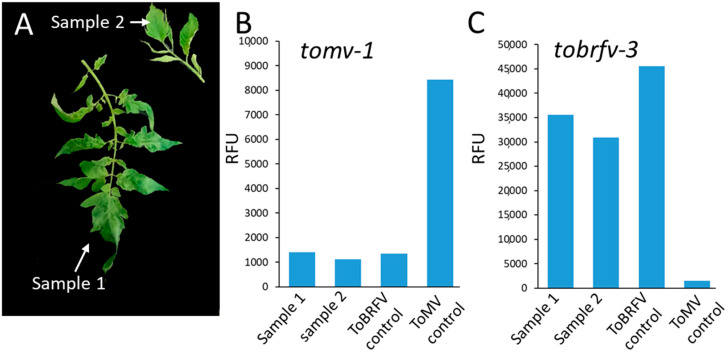
Detection of ToBRFV in field samples using CRISPR/Cas12a. (**A**) Tomato with mosaic leaf pattern were detected in two different sites in Azriel village, Israel, in December 2020. Samples were obtained from two infected tomato cultivars, Ikram and Sgula. (**B**) CRISPR/Cas12a-based detection using the *tomv-1* crRNA indicated no presence of ToMV. (**C**) CRISPR/Cas12a-based detection using the *tobrfv-3* crRNA indicated the presence of ToBRFV in the sample.

Next, we tested the applicability of the CRISPR/Cas12a system for specific detection of tobamoviruses in two field samples from Azriel village, Israel. In two separate greenhouses, plants with severe mosaic and shoestring leaf symptoms were detected, consistent with tobamovirus infection (Figure 4A). We used our CRISPR/Cas12a system to diagnose if the plants were infected with ToMV or ToBRFV. Analysis using ToMV crRNA *tomv-1* revealed that both tomato plants were likely not infected with ToMV, as indicated by the weak fluorescent signal that was similar to the ToBRFV as negative control (Figure 4B). In marked contrast, the ToBRFV crRNA *tobrfv-3* showed high fluorescence for both samples, which was similar to the ToBRFV positive control (Figure 4C). To confirm these results, RT-PCR products from both samples were sequenced. Indeed, both samples showed 100% identity to ToBRFV-IL (KX619418.1) and 76.91% to ToMV (AF332868). These results establish that the CRISPR/Cas12a system can detect ToBRFV in field samples.

## 4. Discussion

ToBRFV is an emerging tobamovirus, which is spreading rapidly throughout the world and poses a substantial threat to the global tomato industry. ToBRFV symptoms are similar to other tobamoviruses, such as ToMV. These challenges drove the development of new real-time PCR and LAMP assays for specific ToBRFV detection [31,48]. While it was shown that CRISPR/Cas12a can be used to detect plant viruses [44], it was unknown if it could be applied to distinguish between closely related plant viruses of the same genus. Here, we provide evidence that the CRISPR/Cas12a technology can also be applied to identify ToBRFV and distinguish it from the closely related ToMV (Figure 2). Using this method, ToBRFV can be detected with low concentrations of RT-PCR products, and also when mixed with ToMV (Figure 3). Detection of ToBRFV in field samples (Figure 4) demonstrates this technology’s potential as a bona fide virus identification method that can be used in agricultural practice.

Further research is required to determine if our method is able to distinguish ToBRFV from solanaceous-infecting viruses other than ToMV. The *tobrfv-3* crRNA sequence is highly specific to ToBRFV, and shows no significant similarity to the closely related viruses TMV (V01408), tomato mottle mosaic virus (ToMMV) (KF477193) and rehmannia mosaic virus (RheMV) (EF375551) according to BLAST analysis. Therefore, it can be anticipated that this CRISPR/Cas12a system will also be able to distinguish between ToBRFV and these tobamoviruses.

While the current setting of our experiment still requires a thermocycler, further development can enhance the CRISPR/Cas12a method for more accessible and user-friendly applications. For example, CRISPR/Cas12a can be combined with isothermal amplification methods such as loop-mediated isothermal amplification (LAMP) or recombinase polymerase amplification (RPA), to detect ToBRFV quickly and accurately in less than 15 min. Such detection platforms named SHERLOCK and DETECTR [42,49] were successfully commercialized for SARS-Cov-2 detection based on CRISPR/Cas13 and Cas12a, respectively. Importantly, such technology can be applied using simple incubation instrumentation, such as a low-cost hand warmer (~USA Dollars 0.3), rather than expensive and complex thermocyclers [50]. Moreover, the LAMP/RPA-coupled CRISPR-Cas12 technology is cost effective, with a price of 2–5 USD/reaction [51], which is comparable to existing RT-PCR and real-time RT-PCR techniques. Our protocol still relies on the RT-PCR step. Replacement of RT-PCR with isothermal amplification such as RPA or LAMP, as in SHERLOCK and DETECTR, would significantly improve the applicability of our system and enable its future application in the field.

Recently, it was shown that the amplification step may be avoided through signal amplification. One such option is to use gold nanoparticles, or L-methionine gold nanoclusters combined with electrochemiluminescence to enhance and stabilize the signal [52,53]. Another approach was recently reported where the use of several crRNAs, targeting several loci within the target DNA, significantly increased the fluorescent signal achieved following Cas13 activation [54]. In addition, several reports have shown that the output signal can be visual rather than fluorescent, further simplifying the detection procedure. Such outputs may be colorimetric, or by using lateral flow strips, similar to common pregnancy tests [42,52]. Collectively, these approaches will allow onsite, rapid, and accurate detection without the need for specialized training or equipment. These techniques will be especially relevant for the detection of closely related pathogen species or strains, and resistance-breaking variants.

## Figures and Tables

**Table 1:** Oligonucleotides used for crRNA synthesis.

Name (* nt Position)	Sequence (5′-3′)
T7-crRNA-F	ATCTACAACAGTAGAAATTCCCTATAGTGAGTCGTATTAATTTC
*tobrfv-3* (3023–3049)	** ACCGACGATGTACACATGACATGatctacaacagtagaaattccctatagtgagtcgtattaatttc
*tobrfv-5* (2826–2852)	GCACAGAGACATAGAAACAAGAAatctacaacagtagaaattccctatagtgagtcgtattaatttc
*tobrfv-9* (1822–1848)	GAGGTAGACCCAATGACTGCAGCatctacaacagtagaaattccctatagtgagtcgtattaatttc
*tomv-1* (2469–2495)	GCCATATCAGAATATACTACCGAatctacaacagtagaaattccctatagtgagtcgtattaatttc
*tomv-7* (2295–2321)	GAAGCAACATCCAGAACTCCGAAatctacaacagtagaaattccctatagtgagtcgtattaatttc
*tomv-8* (1746–1772)	AGTACAGACAGTTCGGACAGTGCatctacaacagtagaaattccctatagtgagtcgtattaatttc
*tomv-9* (2906–2932)	CCGTACCCTGCGCACTTTGCAAAatctacaacagtagaaattccctatagtgagtcgtattaatttc

* Numbers indicate the nucleotide positions of each crRNA binding site on the viral sequence. ** Capital letters mark the DNA-binding part and lower case letters mark the conserved sequence in each crRNA.
